# Exercise-Induced Plasma Metabolomic Profiles in Patients With Peripheral Arterial Disease

**DOI:** 10.3389/fphys.2021.758085

**Published:** 2021-11-18

**Authors:** Wendsèndaté Yves Semporé, Juan Manuel Chao De La Barca, Jeanne Hersant, Nafi Ouédraogo, Téné Marceline Yaméogo, Samir Henni, Pascal Reynier, Pierre Abraham

**Affiliations:** ^1^Centre MURAZ, National Institute of Public Health, Bobo Dioulasso, Burkina Faso; ^2^Sports Medicine Department, University Hospital of Angers, Angers, France; ^3^MitoVasc Research Unit, CNRS 6015, INSERM U-1083, University of Angers, Angers, France; ^4^Biochemistry and Molecular Biology Laboratory, University Hospital of Angers, Angers, France; ^5^Vascular Medicine Department, University Hospital of Angers, Angers, France; ^6^Physiology, Nazi Boni University, Bobo Dioulasso, Burkina Faso; ^7^Internal Medicine, Nazi Boni University, Bobo Dioulasso, Burkina Faso

**Keywords:** walking impairment, exercise, treadmill test, transcutaneous oximetry, metabolomic profile

## Abstract

**Aim:** A better knowledge of the biological consequences in the blood of these exercise-induced ischemic events in lower extremity artery disease (LEAD) may improve the prospects of disease management. We explored the preminus postexercise metabolomic difference in 39 patients with LEAD referred for a treadmill oximetry test [transcutaneous oximetry (TcPO_2_)].

**Methods:** Ischemia was estimated through the sum of decrease from rest of oxygen pressure (DROPs) (limb TcPO_2_ changes minus chest TcPO_2_ changes) at buttocks, thighs, and calves regions. Targeted metabolomic analyses measuring 188 metabolites were performed on a few microliters blood samples taken at the earlobe at rest and 3 min after exercise.

**Results:** Maximum walking distance (MWD) was 290 m (120–652 m) and ankle brachial index (ABI) was 0.67 ± 0.17. Supervised paired partial least squares discriminant analysis based on 23,345 models showed good predictive performance for test sets with a median area under the receiver operating characteristic (AUROC) curve value of 0.99 and a *p*-value of 0.00049. The best discriminant metabolites contributing to the model included a subset of 71 (47%) of the 150 accurately measured metabolites in the plasma, comprising 3 acylcarnitines, 3 amino acids, 5 biogenic amines, 9 sphingomyelin, 7 lysophosphatidylcholines, and 44 phosphatidylcholines. In addition, 16 of these metabolites were found to correlate with one or more severity scores of the LEAD.

**Conclusion:** Our results provide new insights into the biological changes that accompany exercise in LEAD and contribute to a better understanding of walking impairment pathophysiology in LEAD, highlighting new candidate biomarkers.

## Introduction

Lower extremity artery disease (LEAD) is a vascular pathology mainly resulting from atherosclerosis that obstructs arteries and compromises vascularization of the muscles in the lower limbs (Serrano Hernando and Martín Conejero, 2007; Krishnan and Collins, 2014). The prevalence of LEAD increases with age. The number of patients with LEAD worldwide is estimated at more than 200 million (Fowkes et al., 2013). In France, the prevalence of LEAD defined by an ankle brachial index (ABI) less than 0.9 is estimated at 16% in the population of 65 years old (Walters et al., 1992; Boccalon et al., 2000; Criqui and Aboyans, 2015; Fowkes et al., 2017). The overall prevalence of peripheral arterial diseases increased by 25% between 2000 and 2010, especially in low- and middle-income countries (Fowkes et al., 2013). In its early stages, LEAD is characterized by chronic and repeated episodes of transient ischemia during walking due to the insufficient delivery of oxygen to meet the oxygen requirements of lower limb exercising muscles. A better knowledge of the biological consequences of these transient exercise-induced ischemic events is fundamental to developing potential future therapeutic improvements.

Transcutaneous oximetry (TcPO_2_) allows for the evaluation of the severity and diffusion (buttocks, thighs, and calves) of exercise-induced lower limb ischemia (Abraham et al., 2003) and the detection of eventual systemic exercise-induced hypoxemia (Abraham et al., 2018, 2021).

The recent development of omics sciences, in particular metabolomics, permits deep biochemical phenotyping through the detection and quantification of a broad spectrum of metabolites in biological fluids with diagnostic, prognostic, or therapeutic follow-up values. It is increasingly used in the investigation of cardiovascular diseases and has enabled great advances in the knowledge of the biology of ischemia in recent years (Dang et al., 2018; Ismaeel et al., 2019; Schranner et al., 2020).

Previous studies have focused on studying the resting metabolomic profile of patients with LEAD at different stages of the disease (i.e., intermittent claudicants vs. critical limb ischemia) compared with controls. Different metabolomic signatures were found between patients with LEAD and controls with concern to the concentrations of serum amino acids, serum acylcarnitines and hexoses, serum biogenic amines, serum ceramides, serum cholesteryl esters (CEs), sphingomyelins, diglycerides, triglycerides, and phosphatidylcholines (Ismaeel et al., 2019). In another study, Azab et al. (2020) showed the different metabolomic signatures between these different categories of patients with LEAD and control subjects. Ismaeel et al. (2019) also found significant correlations between resting ABI and blood ceramides. Other authors confirmed different resting metabolomic signatures between male patients with LEAD and controls in a comparative metabolomic study on serum low-molecular metabolites and oxidized low-density lipoprotein (oxLDL) (Zagura et al., 2015). To the best of our knowledge, no study investigated the metabolomic signature and its correlation with the severity of exercise-induced ischemia in patients with LEAD.

Our main objective was to determine the metabolomic impact of exercise on blood in a small set of highly selected patients with LEAD by confirming the presence of limb exercise-induced ischemia (with TcPO_2_) and by excluding factors that interfere with biological results (e.g., exercise-induced hypoxemia, non-vascular limitation). Our secondary objective was to evaluate the correlation between metabolomic changes and clinical indices of LEAD severity at rest and exercise in those patients.

## Materials and Methods

### Participants

We conducted a prospective study from October 2017 to March 2019. The protocol was systematically proposed to all the adult (≥18 years old) patients referred to the Vascular Medicine Department of the University Hospital of Angers for a treadmill exercise TcPO_2_ with significant walking limitation [self-reported Walking Estimated Limitation Calculated by History (WELCH) score ≤ 50] for at least 3 months and no self-reported non-limb limitation. Most patients are referred to us for the diagnosis of the vascular origin of exercise-related pain. Non-inclusion criteria were coagulation disorders or on anticoagulant therapies, self-reported pulmonary, osteoarticular, or cardiac limitation, inability to walk on the treadmill at the goal speed (walking time over 10 m at spontaneous speed > 10 s), and participation in another protocol.

Exclusion criteria were the absence of limitation on the treadmill (walking time > 15min), absence of symptoms in the lower limbs, non-vascular limitation to walking (myocardial ischemia and joint pain), systemic hypoxemia induced by exercise defined as a chest decrease from rest > 5 mm Hg during exercise, absence of significant ischemia on all the limb probes, and analytic or blood sample failure or biological outlier values. These strict inclusion/exclusion criteria were expected to result in many excluded patients but in a highly selected population with proof of limiting limb ischemia and absence of non-limb limitation.

### Ethical Approval

This study was approved by the “Ile de France II” personal protection committee on September 8, 2017, under number 00001072. Written informed consent was obtained from each participant before inclusion in this study after a full explanation of the goal and methodology of this study. This study was registered on the clinical trials on September 28, 2017, under number NCT03305198 before first inclusion and performed according to the recommendation of the Declaration of Helsinki.

### Measurements

At inclusion, clinical and anthropometric information were measured or fully extracted from the medical records of the patients including age, weight, height, abdominal circumference, history of diseases, and treatments. Claudication was defined as pain in the lower limbs (calves, thighs, and buttocks) occurring when walking and forcing the patient to stop. ABI was defined by the ratio of the systolic pressure in the legs to the systolic pressure in the arms. LEAD was defined as ABI < 0.9. The maximum walking distance (MWD) and the maximum walking time (MWT) were determined during the treadmill walking test at a constant load of 3.2 kmh^–1^ and a slope of 10%.

### Exercise Oximetry

Exercise TcPO_2_ measurements were performed during a treadmill exercise test under electrocardiographic supervision according to a standardized procedure and set of equipment (PF6000 Perimed^®^, Stockholm, Sweden, United Kingdom) connected to a computer with the software AcqTcPO_2_, which automatically calculates in real time the decrease from rest of oxygen pressure (DROP: limb changes minus chest changes) (Abraham et al., 2005).

We used eight probes (one on each calf, one on each thigh, one on each buttock, and two on the chest). The left thoracic probe was considered a default by the computer as the reference probe for the DROP calculation. In the event of measurement anomalies or failure of the left thoracic TcPO_2_ sensor head, the right thoracic probe was used as a reference probe for the calculation of DROPs. After positioning the probes, we waited between 10 and 20 min for the local temperature of the probes to rise to 44°C and for the transcutaneous pressure values in O_2_ to stabilize. The recording of the TcPO_2_ treadmill exercise test took place in three steps:

– At rest for 2 min, with the patient standing still.
– During exercise: The treadmill speed began at 1 km/h and was progressively increased to a maximum of 3.2 kmh^–1^ in 1 min. This walking speed, with a constant slope of 10%, was maintained until the maximum walking capacity was reached (and not just the appearance of pain) in case of myocardial ischemia (ST depression > 1 mm) or after a maximum of 15 min (absence of limitation).
– Following exercise for 10 min during which the TcPO_2_ measurement was continuously monitored and the patient remained on his or her feet and the treadmill was at rest.

Throughout the test, patients were asked to report limb and non-limb symptoms. At the end of the test, the minimum DROPs of each of the probes at the lower limbs and the MWD were collected. The presence of ischemia was defined as DROP < −15 mmHg (Abraham et al., 2003; Bouyé et al., 2004). The presence of exercise-induced systemic hypoxemia was defined as a decrease in chest TcPO_2_> 5 mmHg (Abraham et al., 2021). For further analysis, the sum of minimal DROPs of the six limb probes was used as an index of LEAD severity.

### Biology

#### Preanalytical Aspects

We performed two blood samples per patient, one at rest before the treadmill test and a second sample at 3 min of rest after exercise. For each sample, 200 μl of blood were collected after an earlobe incision in a glass capillary rinsed with heparin. After homogenization, the glass capillary was immediately centrifuged at 2,000 g for 4 min without brakes and at room temperature. An aliquot of 50–60 μl of the supernatant (plasma) was prepared for each sample in polypropylene tubes and frozen at −20°C before being transferred on ice on the same day to the biochemistry and molecular biology laboratory, where it was stored at −80°C before metabolomic analysis.

#### Analytical Aspects

We used a targeted metabolomics approach by using a standardized kit developed by the Austrian company (Biocrates Life Sciences, Innsbruck, Austria). This kit (AbsoluteIDQ^®^ p180) benefits from internal standards, quality controls, and dedicated analysis software and has been validated by numerous publications in clinical biology. This kit allows for the quantification of 188 endogenous molecules including free carnitine (C0), 39 acylcarnitines (C), the sum of hexoses (H1), 21 amino acids, 21 biogenic amines, and 105 lipids (refer to Supplementary Table 1). Four different classes of lipids are detected by the kits: 14 lysophosphatidylcholines (lysoPC), 38 diacylphosphatidylcholines (PCaa), 38 acyl-alkyl-phosphatidylcholines (PCae), and 15 sphingomyelins (SM). For metabolite identification and quantification, the Q-Trap 5500 Mass spectrometer (AB Sciex LLC, Redwood City, California, United States) coupled with the 1260 Agilent high performance liquid chromatography (HPLC) system and the ECLIPSE XDB-C18 3.5 μm 3.0 mm × 100 mm Column (Agilent Technologies, Santa Clara, California, United States) were used. Flow-injection analysis with tandem mass spectrometry (FIA-MS/MS) was used for quantifying acylcarnitines, glycerophospholipids, sphingolipids, and sugars, whereas liquid chromatography (LC) enabled the separation of amino acids and biogenic amines before detection of LC with tandem mass spectrometry (LC-MS/MS).

All the reagents used in this analysis were of LC-MS grade and purchased from the VWR (Fontenay-sous-Bois, France, United Kingdom) and Merck (Molsheim, France, United Kingdom). Sample preparation and analyses were performed following the Kit User Manual. Each plasma sample was thawed and vortexed thoroughly after thawing and centrifuged at 4°C for 5 min at 5,000 g. A total of 10 μl of each sample were mixed with the isotopically labeled internal standard in a microtiter plate and dried under nitrogen flow (nitrogen evaporator 96 well plate; Stuart SBM 200 D/3, Stuart, Stone, United Kingdom). Metabolites then were derivatized with phenylisothiocyanate (PITC) 5% for 20 min at room temperature and subsequently dried for 30 min under nitrogen flow. For extraction, the first 300 μl extraction solvent (5 mM ammonium acetate in methanol) was added and incubated with shaking at 450 revolutions per min (IKA MS3 digital, Thermo-Fisher Scientific, Illkrich, France, United Kingdom) for 30 min at room temperature followed by filtration by centrifugation (Hettich Zentrifugen Rotina 380R, Bäch, Switzerland, United Kingdom) for 2 min at 500 g. Subsequently, 200 μl were removed from the filtrate, transferred to a fresh microtiter deep-well plate, and diluted with 200 μl water for LC-MS analysis of biogenic amines and amino acids. To the remaining 100 μl from the filtrate, 500 μl of MS running solvent was added for FIA-MS/MS. Both the types of measurement were performed on the QTRAP mass spectrometer applying electrospray ionization (ESI) (AB Sciex API5500Q-TRAP, SCIEX, Villebon-sur-Yvette, France, United Kingdom). The MS was coupled to an HPLC (Agilent Technologies 1200 series, Les Ulis, France, United Kingdom). In the case of LC-MS, the metabolites were separated by a hyphenated reverse phase column (Agilent, Zorbax Eclipse XDB C18, 3.0 mm × 100 mm, 3.5 μm) preceded by a precolumn (Security Guard, Phenomenex, C18, 4 mm × 3 mm, Phenomenex, Torrance, California, United States) applying a gradient of solvent A (formic acid 0.2% in water) and solvent B (formic acid 0.2% in acetonitrile) over 7.3 min (0.5 min 0% B, 5 min 70% B, 0.3 min 70% B, 2 min 0% B) at a flow rate of 500 μl/min. The oven temperature was 50°C. For LC-MS analysis and for FIA, 10 and 2 μl × 20 μl samples, respectively, were subjected to measurements in the positive and negative modes. The identification and quantification were achieved by multiple reaction monitoring (MRM) standardized by applying spiked-in isotopically labeled standards in the positive and negative modes, respectively. For calibration, a calibrator mixture consisting of seven different concentrations was used. Quality controls derived from lyophilized human plasma samples were included for three different concentration levels. For FIA, an isocratic method was used (100% organic running solvent) with varying flow conditions (0 min, 30 μl/min; 1.6 min 30 μl/min; 2.4 min, 200 μl/min; 2.8 min, 200 μl/min; 3 min 30 μl/min). The MS settings were as follows: scan time 0.5 s; IS voltage for positive mode 5,500 V, for negative mode −4,500 V; source temperature 200°C; with nitrogen as the collision gas medium. The corresponding parameters for LC-MS were set as follows: scan time 0.5 s, source temperature 500°C, with nitrogen as the collision gas medium. All the reagents used in the processing and analysis were of LC-MS grade. Milli-Q Water Ultrapure was used fresh after being prepared by the high-purity water system with Millipore (Milli-Di). The raw data of the Analyst software (AB Sciex, Framingham, Massachusetts, United States) were processed by the MetIDQ software, which is an integral part of the p180 Kit (Biocrates Life Sciences AG). This streamlines data analysis by the automated calculation of metabolite concentrations providing quality measurements and quantification.

#### Statistical Analyses

Comparison between included and excluded subjects (among which patients for whom we failed to collect blood samples) was performed by the Mann–Whitney *U*-test or the unpaired *t*-test and the χ^2^-test. After validation of the three levels of quality control used with the kit, the metabolite concentrations were used for statistical analyses only if they were in the quantitation range determined by the calibration curves. Indeed, metabolites with more than 20% of their values outside of the quantitation range (i.e., concentration above the upper limit of quantitation or below the lower limit of quantitation) were not considered. Before removing a given metabolite with more than 20% but less than 40% outside of the quantitation range for statistical analysis, the χ^2^-test was performed with the independence between in/out of range and rest/postexercise conditions as the null hypothesis.

The projection-based multivariate analyses were performed on centered and unit variance scaled data. An unsupervised analysis was conducted by using paired principal component analysis (pPCA) to detect similar groups of samples and outliers, i.e., samples displaying an atypical metabolite profile. Paired partial least squares discriminant analysis (pPLS-DA) was performed to discriminate between rest and postexercise samples on the basis of their metabolomic profiles. The data were randomly divided into the training-validation set (30 samples) and test sets (9 samples) from each group. Samples from the training-validation set were randomly split into 20 samples for training the model and 10 samples used for validation of models built with training samples. From the 30,045,015 possible combinations (i.e., models) obtained by choosing 20 samples from a total of 30, we picked 23,345 combinations by sampling the matrix **C** containing all the combinations on its columns every 1,287 columns. This was done to make the process computationally feasible, considering that the probability that two samples allocated to the same set (training or validation) in a given column of **C** was higher if they were in the same set-in neighbor columns [i.e., columns n and (n + 1) differed only in one element] of **C**. The predictive performance of each of the 23,345 pPLS-DA models built with training samples was evaluated by using the area under the receiver operating characteristic (AUROC) curve value and its associated *p*-value. This *p*-value measures the probability for a given model to randomly predict sample allocation (i.e., rest or postexercise metabolomic profile). Since AUROCs and *p*-values cannot be considered to be normally distributed, median values instead of means were used to evaluate pPLS-DA robustness and to decide whether pPLS-DA models satisfactorily separate the two groups. Cutoff values of 0.8 and 0.05 were selected for the median AUROCs and *p*-values, respectively. Training-validation set strategy allows us to select the best models (i.e., models having the highest AUROC) and eventually evaluate their predictive performance on the test set. The global performance of pPLS models built with training sets was considered satisfactory only if the median AUROC value ≥ 0.8 and the median *p*-value ≤ 0.05. In the case of satisfactory global performance, the selection of the discriminant variable (i.e., metabolites) was based on the variable importance for the projection (VIP) and the loading parameters. VIP values summarize the importance of each variable for the pPLS-DA model, whereas the loading values are indicators of the relationship between the **Y** vector containing the class information (i.e., whether rest or postexercise groups) and variables in the **X** matrix (i.e., metabolites). Variables with a VIP value greater than unity are important for group discrimination. Multivariate analysis was performed by using the mixOmics R package (Kim-Anh et al., 2016).

In addition, we performed the Pearson correlation between the clinical criteria of LEAD severity (ABI, DROPs, MWT, and WELCH score) and metabolite exercise-induced changes.

## Results

**FIGURE 1:**
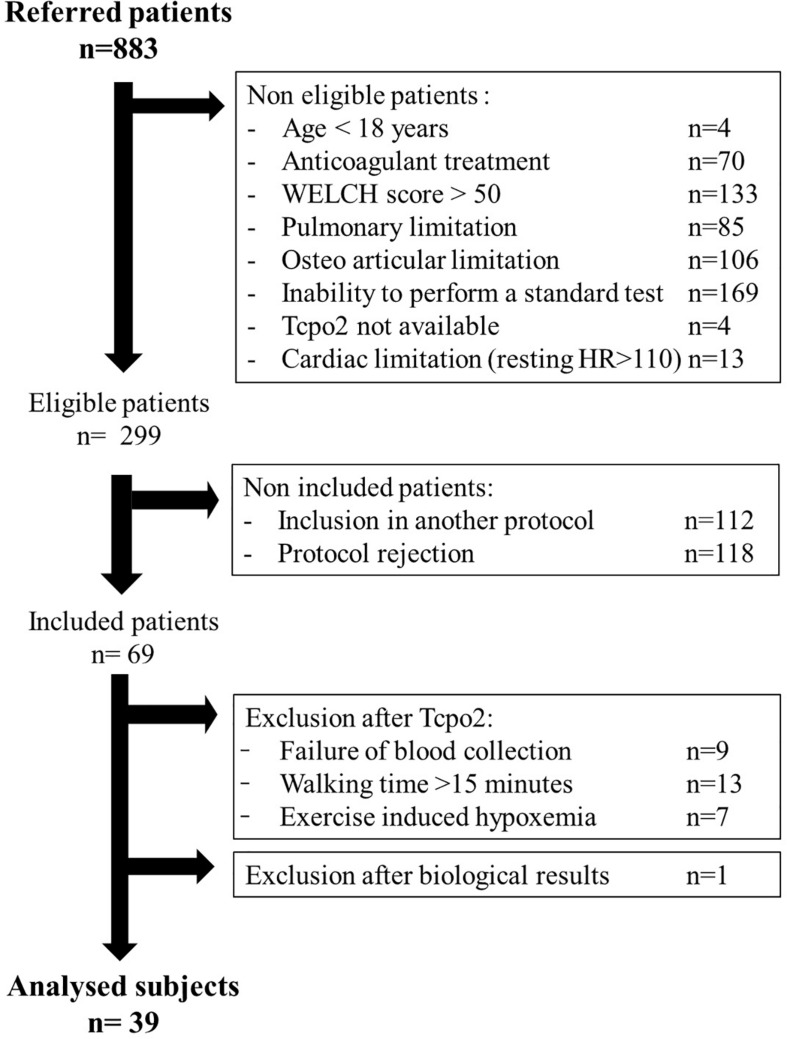
Study flowchart.

From the referred population, we included 69 patients. Of these, 40 patients were available for a metabolomic determination as shown in Figure 1. One patient was excluded after the analysis of blood samples because of outlying results. The general characteristics of 39 analyzed and 30 excluded patients are presented in Table 1. All the patients had a symptomatic LEAD and they reported pain during walking. The majority of patients had a history of hypertension and was a current or former smoker. Almost all the patients were under antiplatelet treatment.

**TABLE 1:** Characteristics of the patients as shown, studied patients were similar to excluded patients except on walking capacity and ankle brachial index (ABI) estimation.

	Analyzed *n* = 39	Excluded *n* = 30	*p*
**Characteristics**			
Age (years)	66.1 ± 11.2	67.3 ± 9.3	0.630
Male gender	34 (87.2)	26 (86.7)	0.950
Weight (Kg)	75.3 ± 13.5	74.1 ± 12.5	0.697
Height (m)	1.68 ± 0.08	1.68 ± 0.07	0.711
Body Mass Index (Kgm^–2^)	26.7 ± 4.4	26.4 ± 4.4	0.829
Ankle Brachial Index	0.67 ± 0.19	0.80 ± 0.23	0.014
WELCH score	18 [10–30]	17 [12–31]	0.771
Hemoglobin concentration (gdcl^–1^)	14.7 ± 1.7	15.1 ± 2.0	0.349
Glycemia (gL^–1^)	1.2 ± 0.4	1.3 ± 0.5	0.349
**History of**			
Hypertension (%)	26 (66.7)	15 (50.0)	0.213
History of lower limb revascularization (%)	20 (51.3)	14 (46.7)	
Diabetes mellitus (%)	5 (12.8)	7 (30.4)	0.253
**Ongoing treatments**			
Antiplatelet treatment (%)	36 (92.3)	30 (100.0)	0.120
Anti-hypertensive drugs (%)	26 (66.7)	15 (50.0)	0.250
Statins (%)	28 (71.8)	25 (83.3)	0.260
Beta blockers (%)	8 (20.5)	9 (30.0)	0.365
**Treadmill test results**			
Maximum walking distance (m)	361 [245–527]	1028 [369–1195]	0.001
Sum of minimal DROP values (mmHg)	103 [84–133]	104 [70–135]	0.517

### Transcutaneous Oximetry

**FIGURE 2:**
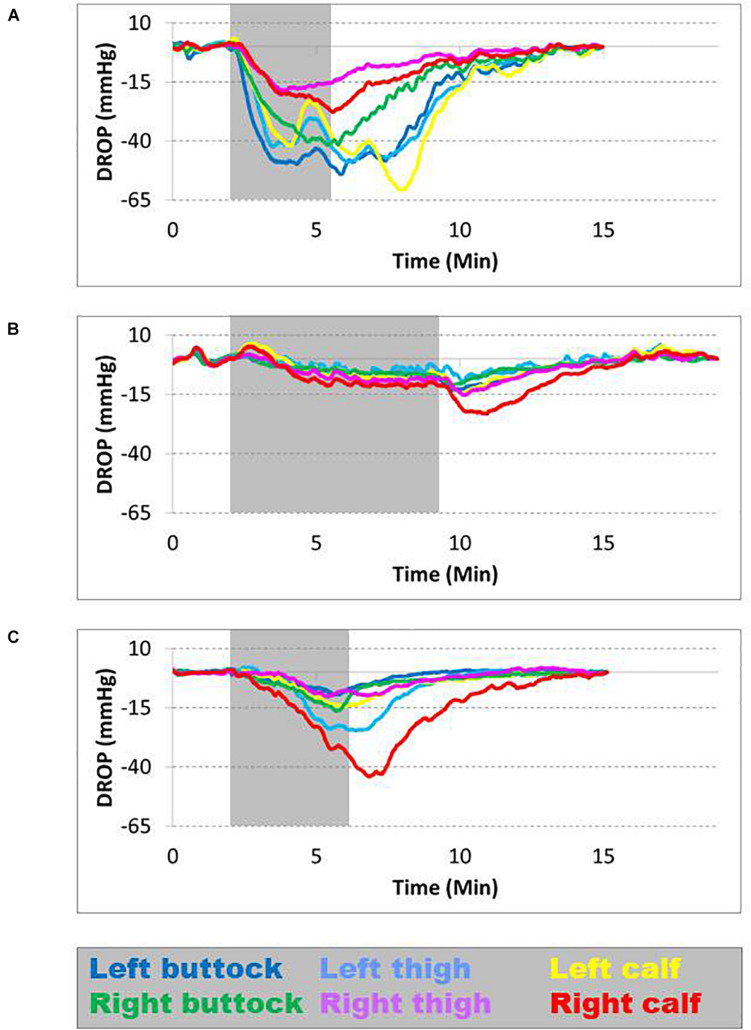
Different types of ischemia found during transcutaneous oximetry (TcPO_2_); each curve represents a specific probe location and is color coded. The gray area represents the period of walking on the treadmill. The normal limit is −15 mm Hg. **(A)** Is patient 15: During walking, all the curves fall below the threshold, indicating ischemia in both the lower limbs. **(B)** Is patient 48: During walking, only the right calf falls below the threshold, indicating an isolated right calf. **(C)** Is patient 52: During walking, ischemia affects predominantly the right calf and left thigh.

**FIGURE 3:**
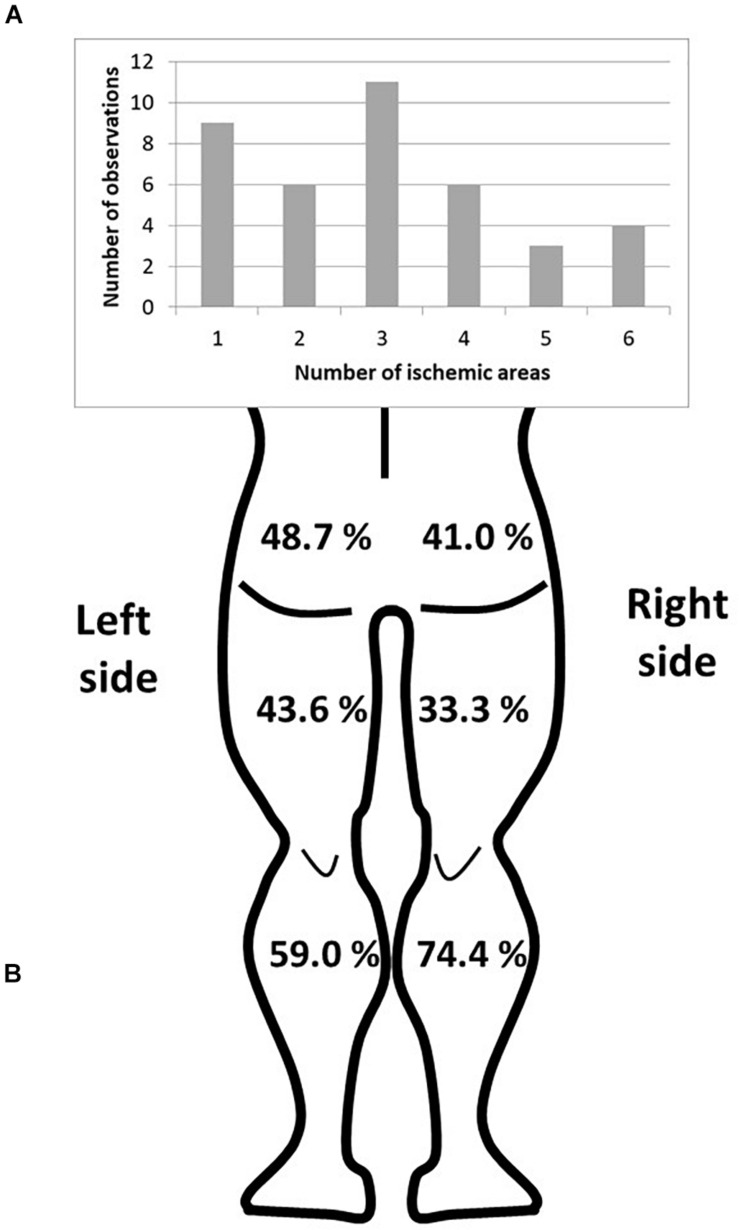
Distribution of the ischemic areas of the lower limbs. **(A)** Number of observations according to the number of ischemic areas. **(B)** Frequency of exercise-induced ischemia by area (buttocks, thighs, and calves) in the lower limbs.

Transcutaneous oximetry was obtained for all the patients, with various degrees of localized (calf only) or diffuses (calves and thighs and buttocks) ischemia. Typical examples of recording are provided as additional content in Supplementary Data (Figure 2). The distribution and frequency of exercise-induced ischemia by area (buttocks, thighs, and calves) in the lower limbs during TcPO_2_ tests are presented in Figure 3. The raw results underlying abnormal individual values after TcPO_2_ tests are presented on a table submitted as Supplementary Table 2.

### Metabolomic Profiles

**FIGURE 4:**
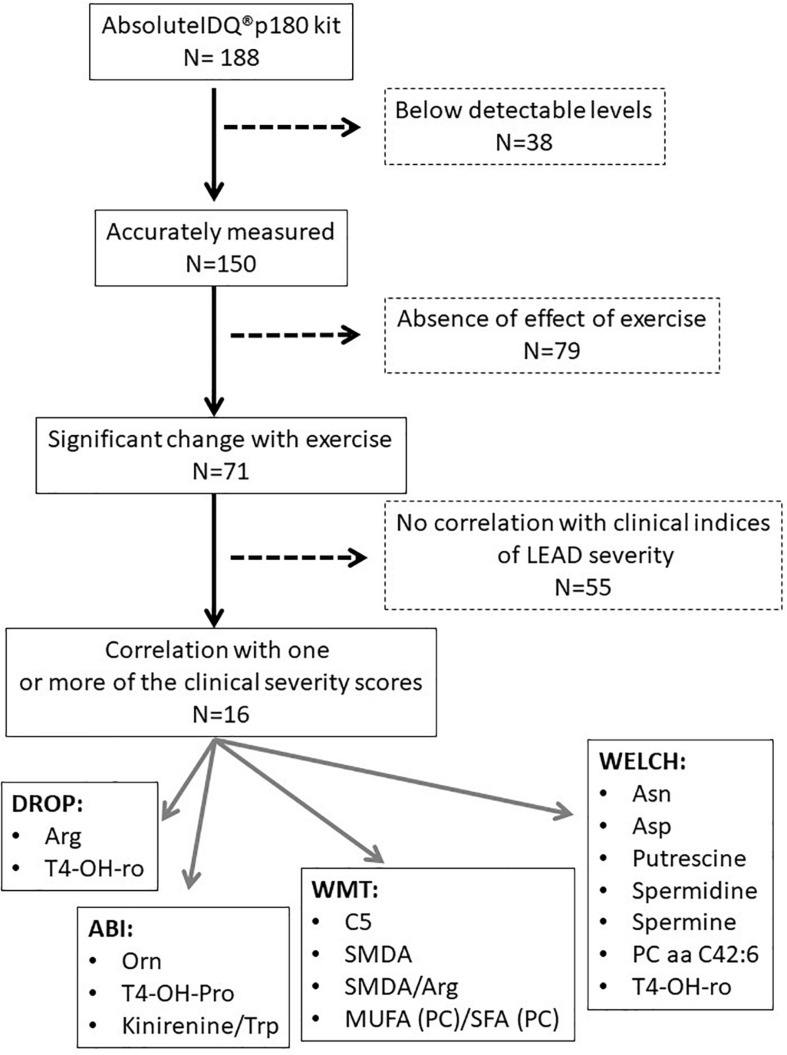
Flowchart of metabolomic analyses.

**FIGURE 5:**
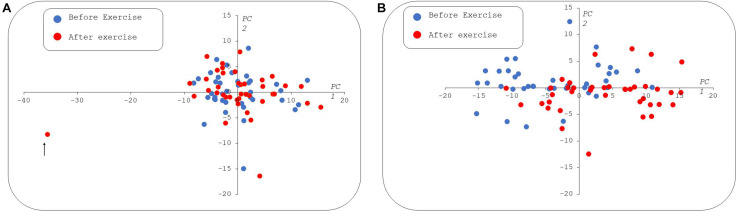
Paired principal component analysis (pPCA) scatter plot of metabolomic data. In the left panel **(A)**, all the samples were used for pPCA, the first principal component (PC1) explains 30% of the total variance and the second (PC2) captures 11%. The sample spotted with a black arrow appears as an outlier in PC1. The right panel **(B)** shows the PCA built without the outlier. In the second pPCA, the first and second PCs explain 43 and 9% of the total variance, respectively. At rest, samples are indicated by blue circles and postexercise samples taken from the same patients as red circles. In both the scatter plots, X and Y axes are dimensionless. PC1, 2: Principal components 1 and 2, respectively.

The flowchart for metabolomic analyses is presented in Figure 4. Among the 188 analyzed metabolites, 150 metabolites were considered accurately measured after validation of the quality controls. These raw data are available in Supplementary Table 3. The non-supervised pPCA approach (Figure 5) highlighted an outlier. The two samples of this patient were excluded from the metabolomics datasets during subsequent statistical analyses.

**FIGURE 6:**
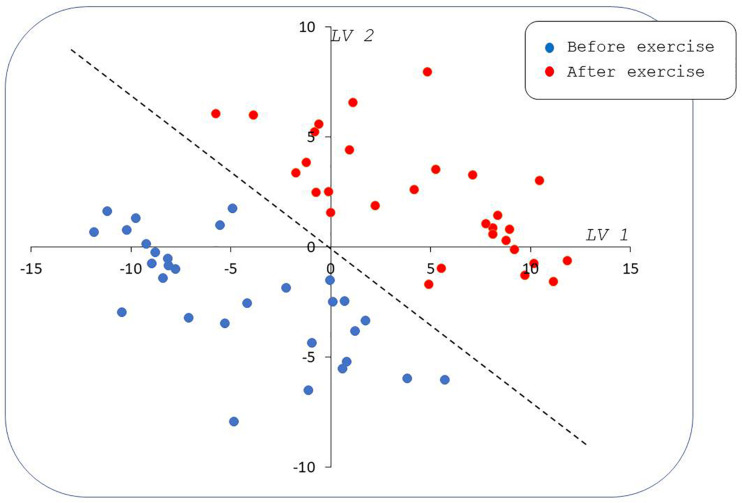
Paired partial least squares discriminant analysis (PLS-DA) scatter plot by using median values for scores of best models. Samples taken from patients before the effort (blue circles) are well separated from samples taken on the same patients after the effort (red circles) in the plane formed by the first and second latent variables (LV1 and LV2). The dashed line represents a frontier between pre- and postexercise samples; this line is a linear combination of LV1 and LV2 indicating an equal contribution of both the latent variables to between-groups discrimination. Note that LV1 and LV2 are dimensionless.

**FIGURE 7:**
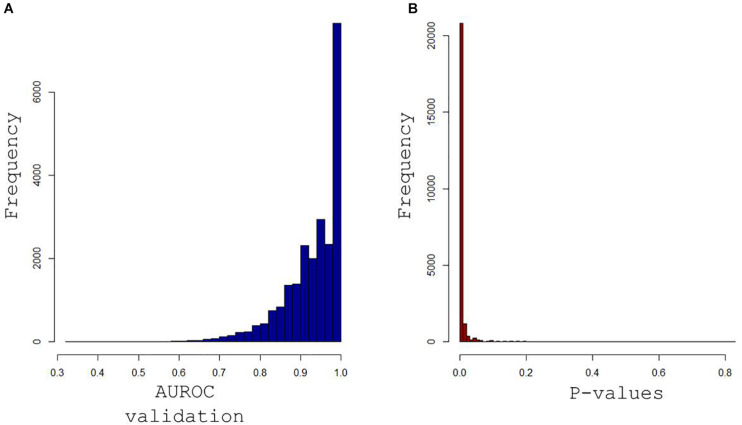
Histograms of the area under receiver operating characteristics (AUROC) curve for validation sets in **(A)** and their associated *p*-values in **(B)** obtained when each of the 23,345 models built with training samples were applied to the remaining validation samples. Median values for the AUROC values and *p*-values (0.95 and 0.00067) demonstrate the excellent predictive capabilities and low overfitting tendency of the paired PLS-DA models.

**FIGURE 8:**
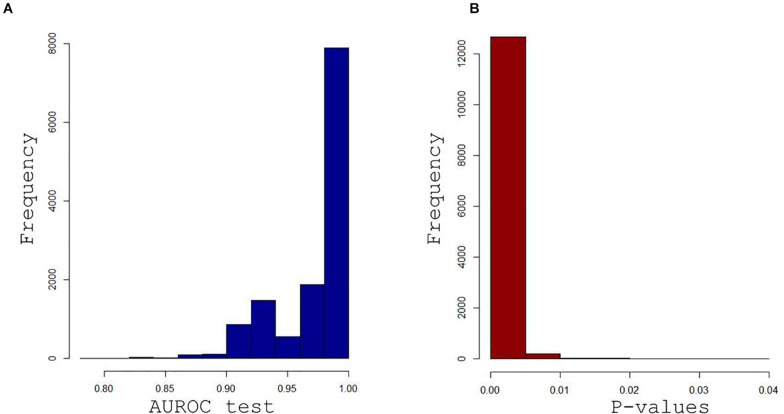
Histograms of the AUROC curve values **(A)** and associated *p*-values **(B)** were obtained when the 12,941 best training-validation models were applied on the test set. Median values for the AUROC values and *p*-values (0.988 and 0.00049) demonstrate the excellent predictive capabilities and low overfitting tendency of the paired PLS-DA best models.

Supervised pPLS-DA based on 23,345 models enabled a clear distinction between rest and postexercise samples (Figure 6) and showed very good predictive performance of training sets on validation sets with a median AUROC value of 0.95 and a *p*-value of 0.00067 (Figure 7). Considering this high global predictive performance, the AUROC cutoff was placed at 0.95 (12,941 models) in choosing the best models among all the 23,345 models. Median values for the AUROC test and *p*-values (0.988 and 0.00049, respectively) demonstrated excellent predictive capabilities and low overfitting of the pPLS-DA best models built with the training sets when applied to the test set (Figure 8).

**FIGURE 9:**
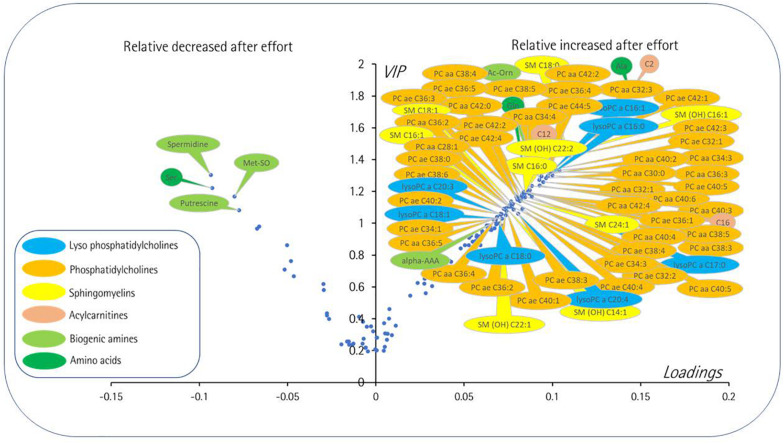
Volcano plot [loadings vs. variable importance for the projection (VIP)] from the 12,941 paired PLS-DA best models comparing at rest and postexercise plasma samples. Only the most important metabolites, i.e., those with VIP ≥ 1, have been labeled. Negative loadings indicate relatively decreased metabolite after exercise compared to the rest situation. The main features are decreased levels after exercise of some polar metabolites including amino acid serine, two polyamines (putrescine and spermidine), and a marker of oxidative stress (methionine sulfoxide). Then again, many lipidic species including diacyl (PC aa) and alkyl-acyl (PC ae) phosphatidylcholines, sphingomyelins (SM), lysophosphatidylcholines (lysoPC), and long chain acylcarnitines (C12 and C16) along with more polar metabolites comprising acetylcarnitine (C2), acetylornithine (Ac-Orn), alpha-aminoadipic acid (alpha-AAA), and amino acids alanine (Ala) and glutamine (Gln). The color coding is as follows: amino acids, dark green; biogenic amines, light green, acylcarnitines, brown; lysophosphatidylcholines, blue; phosphatidylcholines, orange; sphingomyelin, yellow. For phosphatidylcholines, the sum of the length of the two acyl or acyl-alkyl groups is noted after the C and is followed by the number of double bonds. The same notation is used for representing the length and the number of double bonds in the acyl chain of SM and lysoPC.

The best discriminant metabolites (VIP > 1 and high absolute loading values) were combined in a volcano plot (Figure 9). These best discriminant metabolites included a subset of 71 (47%) of the accurately measured metabolites in the plasma, comprising three acylcarnitines (C2, C12, and C16), three amino acids (serine, alanine, and glutamine), five biogenic amines[spermine, putrescine, methionine sulfoxide, acetylornithine, and α-aminoadipate (α-AAA)], nine sphingomyelin, seven lysophosphatidylcholines, and 44 phosphatidylcholines. Four of these metabolites showed postexercise decreased concentrations (spermine, putrescine, serine, and methionine sulfoxide), whereas all the other metabolites had increased concentrations.

A total of 16 of these metabolites, or metabolite ratios, were found to correlate with one or more severity scores (ABI, DROP, MWT, and WELCH scores) of LEAD including kynurenine/tryptophan ratio, hydroxyproline, symmetric dimethyl arginine (SDMA), SDMA/arginine ratio, C5 acylcarnitine, arginine, aspartate, asparagine, lysine, ornithine, proline, putrescine, spermidine, spermine, PC aa C42:6, and the ratio between monounsaturated fatty acid (MUFA) to saturated fatty acid (SFA) or MUFA (PC)/SFA (PC) ratio (Table 2). The summarization of the results of the metabolomic analysis is shown in Figure 4.

**TABLE 2:** Significant correlations between metabolite concentration changes and clinical criteria of severity.

	ABI	*p*-value	DROPs	*p*-value	MWT	*p*-value	WELCH score	*p*-value
C5	–0.083	0.617	–0.143	0.385	0.373	0.020*	0.280	0.085
Arg	0.115	0.487	–0.354	0.027*	0.123	0.454	–0.098	0.554
Asn	0.164	0.318	–0.200	0.221	0.065	0.695	0.319	0.048*
Asp	–0.095	0.563	–0.072	0.665	0.147	0.372	0.400	0.012*
Lys	–0.354	0.027*	0.243	0.136	0.247	0.129	–0.025	0.878
Orn	–0.356	0.026*	0.192	0.241	0.242	0.138	0.121	0.464
Pro	–0.339	0.035*	0.145	0.377	0.121	0.461	–0.053	0.747
Putrescine	0.168	0.307	–0.270	0.097	–0.085	0.607	0.322	0.046*
SDMA	–0.245	0.133	–0.056	0.734	0.385	0.016*	0.047	0.776
Spermidine	–0.121	0.465	–0.053	0.748	–0.030	0.856	0.365	0.022*
Spermine	–0.027	0.871	–0.095	0.567	–0.025	0.878	0.337	0.036*
t4-OH-Pro	–0.404	0.011*	0.327	0.042*	0.225	0.169	–0.211	0.197
PC aa C42:6	–0.286	0.077	0.110	0.507	–0.016	0.924	–0.385	0.015*
SDMA/Arg	–0.232	0.156	0.033	0.844	0.345	0.032*	0.080	0.630
Kynurenine/Trp	0.406	0.010*	–0.114	0.488	0.000	1.000	0.045	0.786
MUFA (PC)/SFA (PC)	0.086	0.603	–0.037	0.822	0.337	0.036*	0.000	0.998

*p < 0.05.

*All other metabolites showed no significant correlations and are not reported in the table. Note that no adjustment was performed for the multiple comparisons.*

## Discussion

While many studies have characterized the exercise-induced metabolomic profiles and have been recently reviewed (Sakaguchi et al., 2019; Morville et al., 2020; Schranner et al., 2020), very few have specifically investigated these profiles in patients with peripheral arterial disease (Ismaeel et al., 2019). At rest in LEAD, there is a known relationship between the metabolomic profile and arterial resistance (Zagura et al., 2015) or the occurrence of early death (Huang et al., 2013). We carried out a comparative study of the metabolomic profile of patients with LEAD by targeting 188 metabolites at rest and after exercise coupled with TcPO_2_ ischemic evaluation. TcPO_2_ has considerably improved the knowledge of LEAD pathophysiology. It allows a better assessment of the severity of LEAD by a quantified measurement of the ischemia (buttock, thigh, and leg) during exercise in a standardized treadmill test. TcPO_2_, therefore, allows us to spatially and temporally quantify exercise-induced ischemia in LEAD (Got, 1998; Abraham et al., 2018). The plasma metabolomic profile of the 150 accurately measured metabolites was sharply affected by exercise in these patients and several metabolites were found significantly correlated with scores of LEAD clinical severity.

### Metabolomic Signature Induced by Exercise in Patients With Lower Extremity Artery Disease

Targeted metabolomics has highlighted a metabolic signature that could improve the prediction of cardiovascular events in the elderly (Rizza et al., 2014). In patients with LEAD, different metabolic signatures exist between patients with intermittent claudication and those with critical limb ischemia (Ismaeel et al., 2019). A relationship between the metabolomic profile and arterial resistance (Zagura et al., 2015) or the occurrence of early death (Huang et al., 2013) was previously observed. In this study, the multivariate analysis revealed strong discrimination after and before exercise, the best discriminant metabolites contributing to the model involving 47% (*n* = 71) of the 150 accurately measured metabolites in the plasma. As far as exercise is a major physiological stress, it is therefore not surprising to find almost half of the metabolites included in the subset of best discriminating metabolites. Four of these metabolites showed postexercise decreased concentrations (spermine, putrescine, serine, and methionine sulfoxide), whereas all the other metabolites (*n* = 67) showed increased concentrations: alanine, glutamine, acetylornithine, α-AAA, C2, C12, C16, nine sphingomyelins, seven lysophosphatidylcholines, and 44 phosphatidylcholines.

The increase in acylcarnitine plasma concentration during exercise is a well-known phenomenon (Sakaguchi et al., 2019; Morville et al., 2020), which reflects either the increased release of these energetic substrates by the liver and adipose tissue to support exercise or their insufficient mitochondrial consumption by skeletal muscle due to exercise-induced ischemia and anoxia. Critical limb ischemia in patients with LEAD (Ismaeel et al., 2019) showed a decreased concentration of acylcarnitines, showing a possible mismatch between acute ischemic attacks and more moderate stress-induced ischemia in this study.

Glucose, the other major energy substrate, is generally increased after exercise (Sakaguchi et al., 2019), but it was found to be lowered after a walking test in patients with intermittent claudication (Coolen et al., 2008). No variation in the glucose level was revealed by our study that accords with the finding of Ismaeel et al. (2019) that compared patients with LEAD to healthy controls at rest (Ismaeel et al., 2019).

The alteration in amino acid metabolism is also a well-known phenomenon during exercise (Sakaguchi et al., 2019; Kelly et al., 2020). However, the modification of branched amino acids (leucine, isoleucine, and valine), which are amino acids linked to insulin resistance frequently reported in postexercise studies (Kelly et al., 2020), was not observed in patients with LEAD. Serine and glutamine, modified after exercise in our patients, have already been shown to be modified in patients with non-LEAD after exercise (Schranner et al., 2020) and might, therefore, not be specific to LEAD. Alanine was one of the most modified metabolites (VIP > 1.8) in our signature. This amino acid is closely related to lactate, which has not been measured here. Its sharp increase is, therefore, evidence that ischemia is induced in patients by exercise.

Methionine sulfoxide (Met-SO) is a biomarker of oxidative stress that we found to be increased after exercise in patients with LEAD. This variation was not found by the two previous studies performed in patients with LEAD at rest (Ismaeel et al., 2019) and after exercise (Coolen et al., 2008) nor after exercise more generally. This metabolite would, therefore, be a potential biomarker of exercise-induced oxidative stress in patients with arteriopathy.

To the best of our knowledge, alpha-aminoadipate is a biomarker of insulin resistance (Wang et al., 2013) that has not been associated with LEAD or with exercise in general. Likewise, the variation observed in this study of acetylornithine has not been previously reported. However, ornithine has been shown to be decreased in peripheral artery disease at rest (Ismaeel et al., 2019). These two metabolites could, therefore, constitute candidate biomarkers of the stress induced by exercise in patients with LEAD.

Little information is available in the literature concerning polyamines (spermine and putrescine), found to be lowered by exercise in this study, in LEAD, or muscular effort in general. An increase in these polyamines has been shown in skeletal muscle in rats during physical exercise (Turchanowa et al., 2000). In contrast to this study, putrescine has been shown to be increased and spermine unmodified at rest in patients with peripheral artery disease (Ismaeel et al., 2019), suggesting a possible involvement of these regulatory molecules in response to the ischemic stress.

A deep remodeling of phospholipids was observed after exercise in patients with LEAD with increased concentrations of 44 phosphatidylcholines, seven lysophosphatidylcholines, and nine sphingomyelins. This probably corresponds to the mobilization of lipoproteins released in the blood to provide energy substrates. This contrasts with the findings of Ismaeel et al. (2019) showing instead that these three phospholipids were not altered at rest in patients with intermittent claudication, but were lowered during episodes of acute lower limb ischemia. Surprisingly, there is very little information available on the effects of exercise on the plasma concentrations of these three phospholipids, and it is difficult to judge the specificity or non-specificity of this phenomenon.

### Metabolites Specifically Related to Ischemia Scores

A total of 16 of these metabolite concentrations was found to correlate with one or more severity scores (ABI, DROP, MWT, and WELCH scores) of the LEAD, including kynurenine/tryptophan ratio, hydroxyproline, SDMA, SDMA/arginine ratio, C5 acylcarnitine, arginine, aspartate, asparagine, lysine, ornithine, proline, putrescine, spermine, spermidine, PC aa C42:6, and the ratio between MUFA to SFA or MUFA (PC)/SFA (PC) ratio.

A large group of these metabolites (SDMA, arginine, ornithine, proline, putrescine, spermine, spermidine, aspartate, asparagine, and lysine) is clearly more or less directly related to the nitric oxide (NO) metabolism, mediating vasodilatation. The correlation of these NO-related metabolites with ischemia is, therefore, not surprising in the situation explored. It should be noted that these metabolites are also often reported as biomarkers of pain, which characterizes the effect of exercise in patients with LEAD. The kynurenine/tryptophan pathway is also involved in vasodilation, pain, and inflammation (Wang et al., 2010).

Hydroxyproline is a metabolite released by the degradation of collagen and marker of tissue damage. Its correlation with the degree of ischemia is, therefore, fully understandable. It is difficult to comment on the correlation of C5 acylcarnitine and PC aa C42:6 with ischemia in the absence of knowledge on the functional specificity of these two lipids compared to their other family members. The MUFA (PC)/SFA (PC) ratio of monounsaturated to saturated glycerophosphocholines is an indicator of fatty acid desaturase activity and may be related to the energetic crisis due to anoxia.

### Limitations

The relatively small sample size may appear as a limitation but in our opinion it is a force of this study to attain a highly selected group with proof of exercise-induced ischemia and absence of non-vascular limitation among which exercise-induced hypoxemia is observed in almost 15% of patients complaining claudication (Abraham et al., 2021). Another limit is the number of metabolites explored by our targeted metabolomics approach. However, the standardized and quantitative nature of this approach provided results close to the quality of clinical biology. The use of capillary samples may appear a limitation but is consistent with the volumes needed for metabolomic analysis with the advantage of being minimally invasive. Last, no control group was studied, but patients were their own control (comparison between postexercise and pre-exercise values). Future studies might be needed to investigate the relationship of biomarkers with new indices of clinical severity of exercise-induced ischemia, such as near-IR spectroscopy, contrast ultrasound, MRI, or muscle scintigraphy (Lindner et al., 2008; Potthast et al., 2009; Pande et al., 2011; Boezeman et al., 2016; Cornelis et al., 2021).

## Conclusion

In the context in which very few metabolomic studies have been carried out in patients with LEAD and even fewer after exercise, this study provides a series of new candidate biomarkers in this pathology. A total of 71 blood metabolites has been shown to be altered by exercise in these patients including metabolites well known to be altered during exercise in general (mainly acylcarnitines and amino acids), and others that were never reported in this context (methionine sulfoxide, α-AAA, acetylornithine, spermine, putrescine, and phospholipid remodeling). A total of 16 metabolites was found to be specifically related to one or more of the indices of clinical severity, two of which correlated to ischemia measured by TcPO_2_. Not surprisingly, a large part of these metabolites is more or less related to the metabolism of NO, which is a major player in vascular response and pain crisis induced by ischemia. Further correlated metabolites reflect the tissue damage (hydroxyproline) and the energy crisis (phospholipids) induced by ischemia. Further studies of these candidate biomarkers are needed to identify the biomarker or the set of biomarkers that may be the most useful in assessing the severity of vascular damage in patients with LEAD.

## Data Availability Statement

The raw data supporting the conclusions of this article will be made available by the authors, without undue reservation.

## Ethics Statement

The studies involving human participants were reviewed and approved by the Comité de Protection des Personnes Ile de France II. The ethics committee waived the requirement of written informed consent for participation.

## Author Contributions

WS, JH, SH, and PA performed the material preparation and data collection. WS, JC, PR, and PA performed the data analysis. PA, JC, and PR conducted the data interpretation. WS wrote the first draft of the manuscript. All the authors contributed to the study conception and design, commented on previous versions of the manuscript, read, and approved the final manuscript.

## Conflict of Interest

The authors declare that the research was conducted in the absence of any commercial or financial relationships that could be construed as a potential conflict of interest.

## Publisher’s Note

All claims expressed in this article are solely those of the authors and do not necessarily represent those of their affiliated organizations, or those of the publisher, the editors and the reviewers. Any product that may be evaluated in this article, or claim that may be made by its manufacturer, is not guaranteed or endorsed by the publisher.

## Supplementary Material

Supplementary Table 1 List of metabolites as provided by the Austrian company Biocrates Life Sciences.

Supplementary Table 2 The raw results underlying abnormal individual values after TcPO_2_ tests.

Supplementary Table 3 Raw data of 150 accurately measured after validation of the quality controls.
