# Supplementary material for: Exercise-Induced Plasma Metabolomic Profiles in Patients With Peripheral Arterial Disease
Source: Front Physiol. 2021 Nov 18;12:758085. doi: 10.3389/fphys.2021.758085 (PMC8637284; doi:10.3389/fphys.2021.758085)
Supplement: Supplementary Table 1 — List of metabolites as provided by the Austrian company Biocrates Life Sciences. [file Table_1.pdf]

List of Metabolites: Absolute*IDQ*® p180 Kit

For detailed information on the composition of the sum signals of glycerophospholipids and sphingomyelins, please refer to the List of Isobars and Isomers for the Absolute*IDQ*® p180 kit.

Amino Acids (21)			
Ala	Alanine	Lys	Lysine
Arg	Arginine	Met	Methionine
Asn	Asparagine	Orn	Ornithine
Asp	Aspartate	Phe	Phenylalanine
Cit	Citrulline	Pro	Proline
Glu	Glutamate	Ser	Serine
Gln	Glutamine	Thr	Threonine
Gly	Glycine	Trp	Tryptophan
His	Histidine	Tyr	Tyrosine
Ile	Isoleucine	Val	Valine
Leu	Leucine		

Biogenic Amines (21)			
Ac-Orn	Acetylornithine	Met-SO	Methionine sulfoxide
alpha-AAA	alpha-Aminoadipic acid	Nitro-Tyr [1)]	Nitrotyrosine
ADMA	Asymmetric dimethylarginine	PEA	Phenylethylamine
Carnosine	Carnosine	Putrescine	Putrescine
Creatinine	Creatinine	Sarcosine	Sarcosine
DOPA	Dihydroxyphenylalanine	Serotonin	Serotonin
Dopamine	Dopamine	Spermidine	Spermidine
Histamine	Histamine	Spermine	Spermine
c4-OH-Pro	*cis*-4-Hydroxyproline	SDMA	Symmetric dimethylarginine
t4-OH-Pro	*trans*-4-Hydroxyproline	Taurine	Taurine
Kynurenine	Kynurenine		

Monosaccharides (1)			
H1	Hexoses (including glucose)		

Acylcarnitines (40)			
C0	Carnitine	C5-M-DC	Methylglutarylcarnitine
C2	Acetylcarnitine	C5-OH (C3-DC-M)	Hydroxyvalerylcarnitine (Methylmalonylcarnitine)
C3	Propionylcarnitine	C5:1	Tiglylcarnitine
C3-OH	Hydroxypropionylcarnitine	C5:1-DC	Glutaconylcarnitine
C3:1	Propenoylcarnitine	C6 (C4:1-DC)	Hexanoylcarnitine (Fumarylcarnitine)
C4	Butyrylcarnitine	C6:1	Hexenoylcarnitine
C4-OH (C3-DC)	Hydroxybutyrylcarnitine (Malonylcarnitine)	C7-DC	Pimeloylcarnitine
C4:1	Butenylcarnitine	C8	Octanoylcarnitine
C5	Valerylcarnitine	C9	Nonaylcarnitine
C5-DC (C6-OH)	Glutarylcarnitine (Hydroxyhexanoylcarnitine)	C10	Decanoylcarnitine

BIOCRATES Life Sciences AG, Eduard-Bodem-Gasse 8, 6020 Innsbruck, Austria, phone: +43.512.579 823, fax: +43.512.579 823-329
email: office@biocrates.com, **www.biocrates.com**, FN 220414p Landesgericht Innsbruck, UID ATU56725128

C10:1	Decenoylcarnitine	C16	Hexadecanoylcarnitine
C10:2	Decadienylcarnitine	C16-OH	Hydroxyhexadecanoylcarnitine
C12	Dodecanoylcarnitine	C16:1	Hexadecenoylcarnitine
C12-DC	Dodecanedioylcarnitine	C16:1-OH	Hydroxyhexadecenoylcarnitine
C12:1	Dodecenoylcarnitine	C16:2	Hexadecadienylcarnitine
C14	Tetradecanoylcarnitine	C16:2-OH	Hydroxyhexadecadienylcarnitine
C14:1	Tetradecenoylcarnitine	C18	Octadecanoylcarnitine
C14:1-OH	Hydroxytetradecenoylcarnitine	C18:1	Octadecenoylcarnitine
C14:2	Tetradecadienylcarnitine	C18:1-OH	Hydroxyoctadecenoylcarnitine
C14:2-OH	Hydroxytetradecadienylcarnitine	C18:2	Octadecadienylcarnitine

Glycerophospholipids (90)			
lysoPC a C14:0	PC aa C34:1	PC aa C42:0	PC ae C38:2
lysoPC a C16:0	PC aa C34:2	PC aa C42:1	PC ae C38:3
lysoPC a C16:1	PC aa C34:3	PC aa C42:2	PC ae C38:4
lysoPC a C17:0	PC aa C34:4	PC aa C42:4	PC ae C38:5
lysoPC a C18:0	PC aa C36:0	PC aa C42:5	PC ae C38:6
lysoPC a C18:1	PC aa C36:1	PC aa C42:6	PC ae C40:1
lysoPC a C18:2	PC aa C36:2	PC ae C30:0	PC ae C40:2
lysoPC a C20:3	PC aa C36:3	PC ae C30:1	PC ae C40:3
lysoPC a C20:4	PC aa C36:4	PC ae C30:2	PC ae C40:4
lysoPC a C24:0	PC aa C36:5	PC ae C32:1	PC ae C40:5
lysoPC a C26:0	PC aa C36:6	PC ae C32:2	PC ae C40:6
lysoPC a C26:1	PC aa C38:0	PC ae C34:0	PC ae C42:0
lysoPC a C28:0	PC aa C38:1 [2)]	PC ae C34:1	PC ae C42:1
lysoPC a C28:1	PC aa C38:3	PC ae C34:2	PC ae C42:2
PC aa C24:0	PC aa C38:4	PC ae C34:3	PC ae C42:3
PC aa C26:0	PC aa C38:5	PC ae C36:0	PC ae C42:4
PC aa C28:1	PC aa C38:6	PC ae C36:1	PC ae C42:5
PC aa C30:0	PC aa C40:1	PC ae C36:2	PC ae C44:3
PC aa C30:2 [2)]	PC aa C40:2	PC ae C36:3	PC ae C44:4
PC aa C32:0	PC aa C40:3	PC ae C36:4	PC ae C44:5
PC aa C32:1	PC aa C40:4	PC ae C36:5	PC ae C44:6
PC aa C32:2	PC aa C40:5	PC ae C38:0	
PC aa C32:3	PC aa C40:6	PC ae C38:1	

Sphingomyelins (15)			
SM (OH) C14:1	SM C18:0	SM (OH) C22:2	SM (OH) C24:1
SM C16:0	SM C18:1	SM C22:3 [2)]	SM C26:0
SM C16:1	SM C20:2	SM C24:0	SM C26:1
SM (OH) C16:1	SM (OH) C22:1	SM C24:1	

[1)] **SCIEX / Waters only**

[2)] **SCIEX only**
