# Supplementary material for: Exercise-Induced Plasma Metabolomic Profiles in Patients With Peripheral Arterial Disease
Source: Front Physiol. 2021 Nov 18;12:758085. doi: 10.3389/fphys.2021.758085 (PMC8637284; doi:10.3389/fphys.2021.758085)
Supplement: Supplementary Table 2 — The raw results underlying abnormal individual values after TcPO2 tests. [file Table_2.pdf]

Patient ID	Left buttock DROPm	Left thigh DROPm	Left calf DROPm	Right buttock DROPm	Right thigh DROPm	Right calf DROPm	Number of ischemic areas
1	**-17**	**-55**	**-64**	**-18**	-13	**-23**	5
2	**-27**	**-18**	-15	**-23**	**-20**	**-44**	5
3	**-31**	-2	-9	**-17**	-5	-3	2
4	**-20**	**-21**	**-35**	-13	**-22**	**-39**	5
5	**-16**	**-17**	**-16**	-10	-13	**-17**	4
6	-4	**-20**	**-36**	-9	-8	**-20**	3
7	**-22**	**-24**	**-35**	**-27**	-12	-14	4
8	-10	-7	-15	**-19**	-8	**-28**	2
9	**-21**	-3	**-16**	**-37**	-13	**-26**	4
10	**-30**	**-33**	**-41**	**-27**	**-20**	**-24**	6
11	**-34**	**-93**	**-64**	**-26**	**-33**	**-27**	6
12	-10	-6	-9	**-28**	**-19**	**-21**	3
15	**-54**	**-49**	**-61**	**-42**	**-19**	**-28**	6
16	**-23**	-15	**-32**	-9	-10	**-16**	3
19	-8	-6	-15	-15	**-18**	**-38**	2
20	**-48**	**-31**	**-37**	**-23**	**-18**	**-17**	6
21	-5	-4	**-32**	-5	-5	-6	1
23	-13	**-27**	**-49**	-13	-12	**-19**	3
27	-10	-14	-12	-9	-12	**-27**	1
29	-11	-4	**-32**	-5	-5	**-26**	2
31	**-36**	-9	-6	**-20**	**-25**	-10	3
32	-3	-8	-4	-3	**-17**	-15	1
33	-11	-3	-10	-7	-3	**-44**	1
36	-13	-9	-13	**-27**	-11	**-16**	2
38	**-20**	**-23**	**-30**	-8	-8	**-18**	4
39	-6	-2	**-16**	-6	-5	-3	1
42	**-25**	**-29**	**-29**	-5	-5	-2	3
34	-4	-6	-7	-6	-7	**-20**	1
45	**-20**	-8	-11	-12	-13	-13	1
48	-13	-10	-15	-11	-15	**-23**	1
52	-9	**-25**	-14	**-16**	-10	**-44**	3
54	-6	-5	-7	-6	-8	**-16**	1
55	**-22**	-10	-15	**-25**	**-19**	**-21**	4
58	**-40**	**-18**	**-46**	-5	-1	-5	3
61	**-19**	**-27**	**-25**	-10	-13	**-26**	4
62	-4	-9	**-19**	-2	**-21**	**-27**	3
65	-8	-3	**-30**	-11	-1	**-40**	2
67	-15	**-42**	**-67**	**-19**	-8	-12	3
69	-13	-15	**-25**	-6	**-17**	**-34**	3
